# Prejudiced interactions: implicit racial bias reduces predictive simulation during joint action with an out-group avatar

**DOI:** 10.1038/srep08507

**Published:** 2015-02-17

**Authors:** Lucia Maria Sacheli, Andrea Christensen, Martin A. Giese, Nick Taubert, Enea Francesco Pavone, Salvatore Maria Aglioti, Matteo Candidi

**Affiliations:** 1 Department of Psychology, Sapienza University of Rome, Italy; 2 IRCCS Fondazione Santa Lucia, Rome, Italy; 3 Section Computational Sensomotorics, Department of Cognitive Neurology, Hertie Institute for Clinical Brain Research; 4 Centre for Integrative Neuroscience, University Clinic Tübingen, Germany

## Abstract

During social interactions people automatically apply stereotypes in order to rapidly categorize others. Racial differences are among the most powerful cues that drive these categorizations and modulate our emotional and cognitive reactivity to others. We investigated whether implicit racial bias may also shape hand kinematics during the execution of realistic joint actions with virtual in- and out-group partners. Caucasian participants were required to perform synchronous imitative or complementary reach-to-grasp movements with avatars that had different skin color (white and black) but showed identical action kinematics. Results demonstrate that stronger visuo-motor interference (indexed here as hand kinematics differences between complementary and imitative actions) emerged: i) when participants were required to predict the partner's action goal in order to on-line adapt their own movements accordingly; ii) during interactions with the in-group partner, indicating the partner's racial membership modulates interactive behaviors. Importantly, the in-group/out-group effect positively correlated with the implicit racial bias of each participant. Thus visuo-motor interference during joint action, likely reflecting predictive embodied simulation of the partner's movements, is affected by cultural inter-individual differences.

As the racial composition of the population changes, intergroup interactions are increasingly common. Social neuroscience is beginning to unravel the ways in which inter-individual differences and cultural factors shape neural and behavioural responses in realistic social contexts^1^. Although implicit in-group preferences may emerge in early childhood^2^, and affect social categorization and evaluations even when processed subliminally^3^,^4^,^5^,^6^, little is known about whether racial bias may also change individuals' behaviour during face-to-face motor interactions.

At a neural level, recent findings show that even basic forms of neurophysiological responses to interpersonal situations such as those typically attributed to simulative mechanisms supported by the activation of the so-called fronto-parietal “mirror system”^7^ are modulated by high-level cognitive and cultural influences. In particular, racial bias induced by the color of the skin of a model may modulate sensorimotor mirroring of observed neutral actions and emotive states^8^,^9^,^10^ as well as the empathic sensorimotor and affective mapping of observed painful stimulation^11^,^12^. Moreover, ethnic categorization modulates neural activations in the human putative “mirror system” during intention understanding^13^ and imitation^14^ (but see also ref. 15). Finally, bodily illusions^5^,^6^ and social attention, as indexed by gaze-mediated orienting^16^, may be influenced by group membership.

Thus, the seemingly automatic tendency to simulate others' sensorimotor states might be reduced when people classify other individuals as “out-group” members. Tellingly, modulation of embodied resonance induced by in-group bias occurs as a function of culturally driven racial prejudices^17^ as it is more prominent in high-prejudice participants and might even disappear in unbiased ones^10^,^11^,^12^.

No study has so far tested whether group bias also modulates face-to-face motor interactions that require individuals to mutually adjust their movements on-line. Studying such situations is crucial for understanding the impact of group bias on action prediction and sensorimotor simulation mechanisms as indirectly indexed by actual movement parameters. Indeed, during joint actions co-agents are required to coordinate with each other without a direct access to the partner's motor plan. Thus, on-line interpersonal coordination necessarily relies on predictions about when a partner will act and what he/she is going to do based on the observation of the behaviour of the other^18^,^19^. Studies suggest that such predictions might be supported by the recruitment of anticipatory simulative fronto-parietal mechanisms^20^,^21^,^22^ which also underlie the on-line monitoring of other's movements^23^,^24^. Importantly, when visual information on the partner's movement is available, “visuo-motor interference”^25^ between self-executed actions and those observed in the partner might also occur. Visuo-motor interference is considered an indirect index of sensorimotor simulation^26^ that, as shown by previous studies^8^,^9^,^10^,^11^,^12^, may be directly influenced by group bias. We hypothesize that: i) group bias modulations of visuo-motor interference effects are more evident under conditions where sensorimotor simulations are essential to predict the goal of the others' movements compared to when this prediction is unnecessary to one's own action fulfillment; ii) these sensorimotor simulations are captured in movement kinematics. Finding these results would suggest that in-group/out-group modulation of sensorimotor simulation in joint action is linked to action prediction.

Here we sought to determine whether movement kinematics and individuals' ability to coordinate with in-/out-group avatars during realistic motor interactions are modulated by individuals' racial bias. To this aim, we asked a group of Caucasian participants to coordinate their reach-to-grasp movements with in-group (Caucasian) or out-group (African) virtual partners that embodied identical real human kinematics. Participants were required to grasp a bottle-shaped object via either a power or a precision grip by on-line adapting to an avatar performing the same or a complementary movement in front of them. Note that precision grip kinematics is sensitive to the cognitive^27^,^28^ and social context in which actions are performed (e.g. the partner's cooperative or competitive intention^29^,^30^,^31^; see also ref. 32 for a review). This higher sensitivity is possibly due to the complex cortical organization needed to control the execution of precision grip movements with respect to power grip ones^33^,^34^ (see also refs. 35,36 for a review). The task included two interactive conditions requiring participants to either adapt only the timing of their movements in order to synchronize with the avatar (Synchronization, Syn) or to synchronize plus on-line select their action goal according to the avatar's movement, in terms of to-be-grasped object part and consequent hand configuration (Joint Action, JA). In JA, participants were not directly informed about which part of the bottle-shaped object they had to grasp (i.e. the upper part with a precision grip or the lower part with a power grip) but were required to perform either imitative (i.e. congruent, precision-precision or power-power grip) or complementary actions (i.e. incongruent precision-power grip, or vice-versa) with respect to the avatar's ones.

We specifically aimed to investigate whether: 1) previous findings on the in-group/out-group modulation of sensorimotor simulation evoked by the observation of others' sensorimotor states might be also measured in the kinematics of face-to-face interactions (as indexed by visuo-motor interference effects); 2) these modulations are unspecific or selectively linked to predictions of others' action goals. These questions were operationalized as follows. On the one hand, the comparison between complementary and imitative interactions would indicate the emergence of visuo-motor interference: indeed, while in imitative actions self-executed movements and those observed in the partner are congruent (and are thus characterized by similar kinematic features), during complementary actions self-executed movements are incongruent with those observed in the partner. Thus, in complementary conditions involuntary simulation of the observed action (manifesting as visuo-motor inference) can be indexed by higher similarity between the executed action and the incongruent one performed by the virtual partner. One may even suggest that any variation in kinematics between imitative and complementary actions which goes in the direction of making the participant's movement features more similar to the partner's one is due to the involuntary simulation of the partner's movements. On the other hand, the comparison between interactions based on prediction of action timing and goal (JA condition) and prediction of action timing only (Syn condition) would capture the impact on participants' motor behaviour of predictions regarding others' action goals (namely, the impact of the need to predict not only the instant at which the avatar grasped the bottle but also the grasped object part, i.e. its action goal). Thus, we expected racial bias would modulate visuo-motor interference - as indexed by the comparisons between complementary and imitative action kinematics - when participants are required to predict the partner's action goal in order to adapt to it (i.e. in JA). This modulation might be more likely to emerge during precision grips as this movement is sensitive to high-level cognitive manipulations^27^,^28^. The analysis of kinematics allowed us to: i) monitor participants' behaviour during the interaction and verify they were on-line adapting to the avatar's movements, thus validating human-avatar interactions as reliable set-ups to simulate human-human joint actions in a controlled environment; ii) measure visuo-motor interference effects through direct investigation of motor behaviour, thus expanding influential studies^37^,^38^,^39^ based on inferences derived from the analysis of response times.

## Methods

### Participants

Fourteen Caucasian participants (9 males, average age 23 ± 2.96 standard deviations) took part in the experiment. The experimental protocol was approved by the ethics committee of the Fondazione Santa Lucia and was carried out in accordance with the ethical standards of the 1964 Declaration of Helsinki. All participants were right-handed as confirmed by the Standard Handedness Inventory^40^, reported normal or corrected-to-normal vision and were naive as to the purpose of the experiment; they gave their written informed consent to take part in the study, received reimbursement for their participation and were debriefed on the purpose of the experiment at the end of the experimental procedure. Anonymized data is available upon individual request and in accordance with the local ethical committee's guidelines.

### Stimuli and apparatus

**Figure 1:**
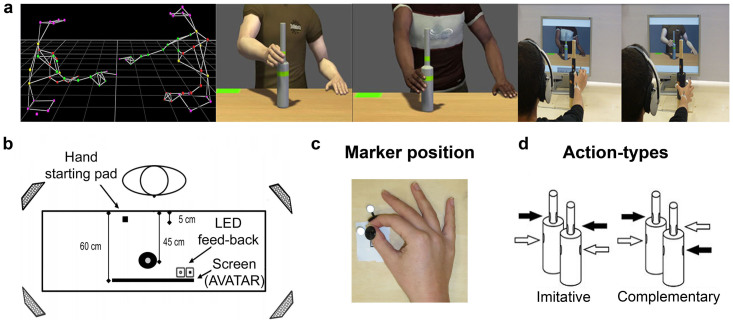
The figure illustrates (a) the steps followed to create the clips of the avatar's movements; kinematics of the virtual partners were recorded using a Vicon MX optical tracking system (Vicon Motion Systems, Oxford, UK) and the final processed trajectories were animated using commercial software (Autodesk, Motion Builder); (b) the experimental set-up; (c) the position of the markers on participants' thumb and index finger of the right hand placed on the table in the starting position with thumb and index finger gently opposed; and (d) a schematic representation of Action-types (Complementary/Imitative).

Participants were comfortably seated in front of a rectangular 120 × 100 cm table watching a 1024 × 768 resolution LCD monitor placed 60 cm from their eyes (see Figure 1, panel b). They had to reach-and-grasp two pairs of touch sensitive copper plates placed at 15 cm (lower location) and 23 cm (higher location) on a bottle-shaped object located 5 cm right from the midline of the working surface, 45 cm away from the participants. The bottle-shaped object (30 cm total height) was constituted by two superimposed cylinders with different diameters (small, 2.5 cm; large, 7.0 cm). Given the object dimension, grasping the lower location would imply performing a power grip while grasping the higher location would imply a precision grip. Before each trial, participants positioned their right hand on a starting button placed at a distance of 40 cm from the bottle-shaped object and 10 cm on the right of the midline, with the index finger and the thumb gently opposed (Figure 1, panel c).

Auditory instructions concerning the movement to be executed were delivered to participants prior to each trial via headphones. They consisted of three sounds having the same intensity (4 db) and duration (200 ms) but different frequency: i) “high-pitched”, 1479 Hz, ii) “low-pitched”, 115.5 Hz, iii) “whistle”, 787.5 Hz. Feedback signals about participants' performance were provided via a green/red LED placed next to the left corner of the screen.

Infrared reflective markers (5 mm diameter) were attached to participants' right upper limb on the following points: i) thumb, ulnar side of the nail; and ii) index finger, radial side of the nail, in order to monitor movement kinematics during the task with a SMART-D motion capture system (Bioengineering Technology & Systems [B|T|S]). The system consisted in four infrared cameras (sampling rate 100 Hz) with wide-angle lenses (placed about 100 cm away from each of the four corners of the table) capturing the movement of the markers in 3D space. The standard deviation of the reconstruction error was always lower than 0.5 mm for the three axes.

The kinematics of the virtual partners was based on the reconstruction of the movements of human participants performing grasping movements in the very same experimental set-up of the present study (see Figure 1, panel a). Each clip showed only the upper body of the avatar from the shoulders to hips, without the neck and the head, and included the avatar's bottle-shaped object. Both the in-group (Caucasian) and the out-group (African) avatar performed the same 12 power and 12 precision grips towards the bottle-shaped object. It is worth noting that the avatar exhibited specific kinematics patterns reflecting precision and power grips. More specifically, power vs. precision grips differed significantly both in terms of maximum grip aperture (Power 181.53 ± 30.76 mm vs. Precision 156.09 ± 16.67 mm, *p* = .005) and maximum height of the wrist (Power 224.05 ± 36.79 mm vs. Precision 235.29 ± 9.83 mm, *p* = .001, note that power grips correspond to grasping the bottle on a lower location and precision grip on a higher location). Crucially, in 33% of the trials, the video included an on-line correction, i.e. the avatar switched from a power to a precision grip (or vice-versa) during the reaching phase. Precision and power grip stimuli did not differ in terms of velocity both in corrections and no-corrections (*p* = .23, *p* = .49). Kinematics of the virtual partners were recorded using a Vicon MX optical tracking system (Vicon Motion Systems, Oxford, UK) with 10 infrared light emitting cameras. 3D positions of 37 passive reflecting markers, attached to the subject's complete upper body (pelvis, chest, head, left and right arm, right hand) were recorded with a spatial error below 1.5 mm and at a temporal resolution of 120 Hz. Raw data were processed offline using commercial Vicon software and the final processed trajectories were animated using commercial software (Autodesk, Motion Builder).

Data was filtered using commercial computer graphics software (Motion builder, 3D Studio Max) that allowed us to avoid artefacts and to minimally distort the appearance of the movement in the original motion capture data.

### Procedure

The experiment was divided in three phases: i) the Implicit Association Test (IAT), ii) the human-avatar motor interaction, and iii) the evaluation of the avatars.

#### Implicit Association Test (IAT)

Participants completed a computerized version of the two-category skin-color IAT^41^,^42^ in order to evaluate their implicit race-related attitude. The IAT provides a measure of the strength of the automatic association between different concepts in memory. In particular, the skin-color IAT measures the relative ease with which participants make associations between Caucasian and African faces and the concepts of good and bad. Easier pairings (i.e. faster responses) are interpreted as being more strongly associated in memory than more difficult pairings (i.e. slower responses). For example, faster responses when white and positive (and black and negative) concepts are paired than when black and positive (and white and negative) concepts are paired reflect a bias for positive associations with white individuals and/or a bias for negative association with black individuals. In the present study, in order to avoid any explicit reference to ethnic bias, the test was introduced to participants as a test on the “velocity of learning associations between visual and verbal stimuli”. Data were analyzed using the improved IAT scoring algorithm recommended by Greenwald et al.^42^, where D scores greater than zero suggest the presence of implicit racial bias, which is instead considered to be absent when D scores are equal to 0. Participants' D-score ranged from 0.16 to 0.92 (m = 0.59 ± 0.3).

#### Instructions and human-avatar motor interaction

Participants were told they would perform a motor task with two different partners whose kinematics had been previously recorded and transferred on different avatars. Written instructions specified participants would watch clips showing an avatar performing reach-to-grasp movements towards a bottle-shaped object; they were shown a (fake) picture of two individuals – with covered eyes and a neutral facial expression - who resembled a Caucasian (“Luca”) and African (“Ibrahim”) student who attended the university in the city where participants lived (Rome); they were told that Ibrahim's and Luca's movements had been implemented in the avatar in order to control for differences in the body shape, but that they could recognize the two participants from the avatar's skin color (white for Luca and black for Ibrahim).

Participants were required to grasp the bottle-shaped object placed in front of them as synchronously as possible with their virtual partner and perform complementary or imitative movements with respect to that of the avatar in two interactive conditions:

1)  in Synchronization (Syn), a high-/low-pitched sound was delivered to signal which part of the object they had to grasp (low-pitched meaning “grasp the lower part” with a power grip, high-pitched meaning “grasp the upper part” with a precision grip). Thus, participants had to adapt the timing of their own movements on the avatar's one in order to achieve the best performance. Note that in this condition instructions led participants to perform complementary/imitative movements with respect to the avatar's ones without any explicit reference to this aspect of the interaction;

2)  in Joint Action (JA), a whistle signaled participants to on-line adapt to the partner's movements. However no beforehand information on which part of the bottle-shaped object they had to grasp (i.e. the upper part with a precision grip or the lower part with a power grip) was provided. Also, participants were required (in different sessions) to perform either imitative (i.e. congruent) or complementary (i.e. incongruent) movements with respect to the avatar's ones. The Complementary/Imitative instruction was given at the beginning of each session. Thus, in JA participants were required to on-line adapt to the avatar both their action timing and goal.

The trial time-line was as follows. First, a fixation cross placed on the region of the screen where the avatar's hand would appear alerted participants about the impending trial. Then, participants heard the auditory instruction (i.e. high/low-pitched sound or whistle), and after 300 ms the clip started. Upon receiving the auditory instruction participants could release the Start-button and reach-to-grasp the object. In the case the participant started before hearing the instruction, the trial was classified as a false-start and discarded from the analyses. At the end of each trial, participants received a feedback (a green/red LED) about their performance (win/loss trial). A win trial implied that participants followed their instructions and achieved synchrony with the avatar in grasping the objects, i.e. when the time-delay between participant's and avatar's index-thumb contact-times on their bottle fell within a given time-window which was narrowed or enlarged on a trial by trial basis according to a stair-case procedure. Namely, the time-window for considering synchronous a given grasp became shorter as participants got better in the task and longer if they failed in three consecutive trials; this procedure allowed tailoring the time-window to assess synchrony on the specific skill of each participant. The avatar's index-thumb contact-times were measured trial-by-trial by a photodiode placed on the screen that sent a TTL signal which was recorded by E-Prime2 software (Psychology Software Tools Inc., Pittsburgh, PA). The photodiode was triggered by a black dot (not visible to the participants) placed on the screen on the frame of the clip corresponding to the moment at which the avatar grasped his virtual object. Previous to any recording of the motor task, participants could listen to the auditory instructions as long as they needed to achieve an errorless association of whistle/high-pitched/low-pitched sounds with the correct instruction; no familiarization block was provided.

Participants performed three Complementary and three Imitative sessions (counterbalanced between participants), each comprising 4 blocks of 24 trials each. Each block comprised two mini-blocks of 12 Syn/JA trials (counterbalanced between participants), where the type of grip (power/precision grip) that participants were required to perform was randomized. The Complementary/Imitative instruction to be followed during JA was given at the beginning of each session. Unbeknownst to the participants, this instruction implied consistent imitative or complementary actions also during Syn condition. Within each session, participants interacted both with the in-group and out-group partner in different blocks (in-group/out-group block order was counterbalanced between participants). To provide an example, a Complementary session might be divided as follows: Complementary-Syn-Ingroup, Complementary-JA-Ingroup, Complementary-Syn-Outgroup, Complementary-JA-Outgroup. Thus, in each session participants watched twice the 24 clips depicting actions performed by the in-group and twice the 24 clips depicting actions performed by the out-group, once during Syn and once during JA. Overall, half of trials required participants to perform a precision/power grip, equally distributed within the blocks. In 33% of the clips, the avatar performed a movement correction (“Correction” clips). This condition was added in order to verify whether in JA participants performed movement corrections in response to movement corrections observed in the avatar. Finding corrections would demonstrate that in JA participants on-line adapt their goals (and, consequently, their grips) to the avatar movements (possibly through action prediction). Stimuli presentation and randomization were controlled by E-Prime2 software (Psychology Software Tools Inc., Pittsburgh, PA). The SMART-D software package (B|T|S|) was used to provide a 3-D reconstruction of the marker positions as a function of time and to analyze hand kinematics.

#### Evaluation of the avatars

At the end of the experiment, participants were asked to rate on a series of Visual Analogue Scales (VAS, 100 mm) different features of the clips showing the avatars' movements. In particular, participants rated the performance achieved during the interaction with the in-group avatar (In-group PERFORMANCE, “Was it easy to coordinate with Luca?”, 100 = very easy, 0 = not easy at all), and with the out-group avatar (Out-group PERFORMANCE, “Was it easy to coordinate with Ibrahim?”, 100 = very easy, 0 = not easy at all). Moreover, they judged the realism of the avatar's movements (REALISM, “Were the movements you observed realistic?” 100 = very realistic, 0 = not realistic). Finally, participants were asked whether they doubted the movements had been recorded from two different individuals, “Luca and Ibrahim” (DOUBT, “Did you doubt the movements belonged to two different people, namely Luca and Ibrahim?”, 100 = I doubted very much, 0 = I did not doubt at all).

### Data handling and design

Only correct trials were entered in the behavioural and kinematics analyses, i.e. we excluded from the analyses trials in which participants i) missed the touch-sensitive copper-plates and response was thus not recorded, ii) made false-starts, or iii) did not respect their auditory instructions and thus grasped the wrong part of the bottle-shaped object, i.e. incorrect trials (total percentage of excluded trials = 9.5 ± 4.5%).

We analyzed as dependent measures:

1. **Accuracy**, i.e. percentage of movements executed according to trial instructions;
2. **Reaction Times** (RTs), i.e. time from the instant when participants heard the auditory instruction to the instant they released the Start-button;
3. **Grasping Asynchrony** (GAsynchr), i.e. absolute value of time delay between the participant' and the avatar's index-thumb contact-times on the bottle, i.e. [abs (participant's contact-time on the bottle – avatar's contact-time on the bottle)];
4. **Maximum grip Aperture** (MaxAp), i.e. the peak of index-thumb 3D Euclidean distance measured during the reach-to-grasp phase. We selected maximum grip aperture kinematics because it is sensitive to the ultimate goal of the grasping and to the social context^27^,^28^,^32^,^35^,^36^,^43^. On the base of these studies, we also expected precision grip trials would be more sensitive to our in-/out-group manipulation than power grip ones. In particular, we analyzed hand kinematics as an indirect index of the recruitment of sensorimotor simulative mechanisms^25^,^26^. We were primarily interested in the comparison between imitative and complementary actions as this comparison may index the extent to which individuals resort to the simulation of the observed movement in order to smoothly coordinate with it. Indeed, any variation in the kinematic pattern between imitative and complementary actions that goes in the direction of making the participant's movement features more similar to the partner's one is likely due to the involuntary simulation of the partner's action. For example, MaxAp should be larger in complementary precision grips (i.e. when the participants are observing the partner performing a large power grip) than in imitative ones (i.e. when the participants are observing the partner performing a small precision grip). Thus, we measured how the tendency to simulate the movements of a partner (i.e. presence of a larger MaxAp in precision grips during complementary as compared to imitative actions) is modulated by task demands (JA and Syn) and by the partner's group membership (in-group/out-group).

For each of the above-mentioned measures we calculated the individual mean in each condition, excluding each value that fell 2.5 SDs above or below each individual mean for each experimental condition as outlier value (on average, 0.6 ± 0.5% of total, namely 3.5 ± 3.2 trials).

**Figure 2:**
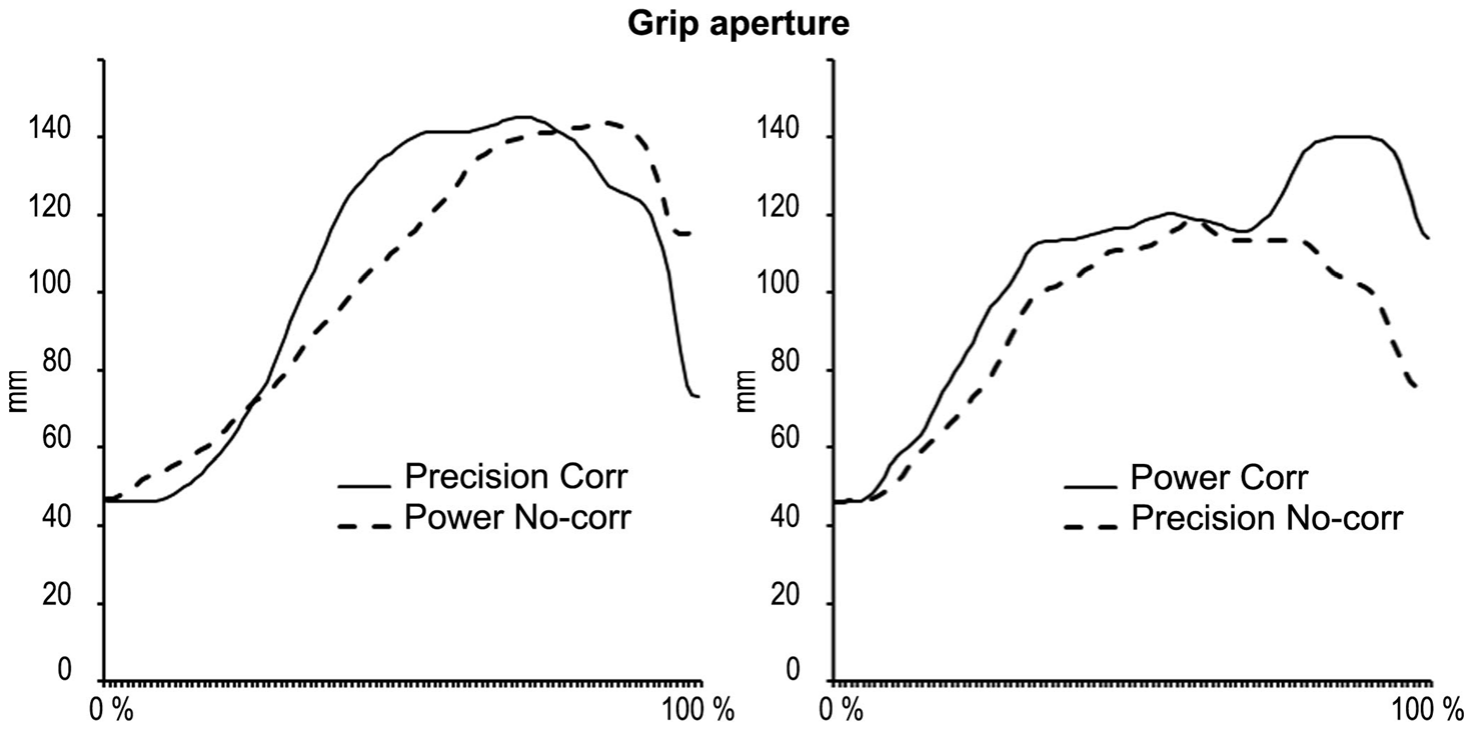
The graph illustrates the shape of grip aperture stereotypical patterns (taken from single tracks of one participant) and shows that in the JA condition correction trials (THICK LINES) showed a similar shape compared to no-correction (DASHED LINES) trials until the closure phase, and then they diverged, thus highlighting that participants on-line adapted to the avatar's movement. Grip aperture data have been normalized on movement time so that the abscissa reports percentages of movement. On the left panel, the kinematic profile of grip aperture in corrections from power to precision grip (THICK LINE): it shows that the participant firstly opened the hand as to perform a power grip during the opening phase, e.g. their grip aperture was similar to a power grip (see DASHED LINE in this panel), and then reduced the aperture so as to match the kinematics of a precision grip during the closure phase (i.e. the final grip aperture is equal to the one of a precision grip, see dashed line in the right panel). Conversely, on the right panel, the figure illustrates grip aperture data in corrections from precision to power grip (THICK LINE): it shows that participants firstly opened their hand as to perform a precision grip, e.g. their grip aperture was similar to a precision grip (see DASHED LINE in this panel), and they then corrected their movement until it matched the aperture of the power grip (i.e. the final grip aperture is equal to the one of a power grip, see dashed line in the left panel).

Raw RTs, Accuracy, GAsynchr and MaxAp individual means were entered in separate preliminary within-subject ANOVAs having Group (In-/Out-group) x Action-type (Complementary/Imitative) x Correction (Correction/No-correction) x Interaction-type (JA/Syn) x Grip-type (Power/Precision grip) as within subjects factors (see Supplementary data). This analysis allowed to preliminarily explore whether the human-avatar interactive scenario is a reliable proxy to human-human joint actions in a controlled environment. We verified whether participants were truly on-line adapting to the avatar's movements during JA, as shown by Figure 2 (see also Supplementary data for an extensive description of significant effects). Moreover, we verified whether the Group factor had an effect on RTs and Accuracy, and suggest that the lack of differences between In-group and Out-group interactions in both the analyses on Accuracy and Reaction Times would indicate that Group modulations on kinematics are unlikely to be accounted for by general perceptual/attentional factors or by speed-accuracy trade-offs.

As to the in-group/out-group effect, we specifically aimed to investigate: i) whether previous findings on the in-group/out-group modulation of sensorimotor simulation evoked by the observation of others' action/pain^8^,^9^,^10^,^11^,^12^ can be also measured in the kinematics of face-to-face interactions, and ii) whether these modulations are selectively linked to action prediction. Thus, our design allows us to explore three important phenomena, namely: 1) the emergence of visuo-motor interference during the interaction (indexed by the comparison between complementary and imitative conditions), 2) the impact on participants' behavior of the need to make reliable predictions about the goal of the partner's movements (indexed by comparison between JA and Syn conditions); and 3) the in-group/out-group modulation of the complementary/imitative x JA/Syn interaction.

We directly tested these phenomena by reducing the GAsynchr and the MaxAp data based on the preliminary ANOVAs (i.e. taking into account the measures that showed Group effects in the preliminary analyses on raw data) and dividing the Complementary by the Imitative condition. All the results concerning these indexes will thus represent visuo-motor interference effects. More specifically, we wanted to directly test the impact of Group membership on visuo-motor interference between self-executed movements and those observed in the partner during complementary actions, and whether this interference had an impact on GAsynchr. Note that while the MaxAp captures visuo-motor interference in kinematics, GAsynchr is necessary for checking whether in-group/out-group modulation of interference had an impact on the ability to coordinate with the avatar. Thus, we performed ANOVAs with Group (In-group/Out-group) x Correction (Correction/No-correction) x Interaction-type (JA/Syn) x Grip-type (Power/Precision) as within subjects factors. RTs and Accuracy of responses were analysed only in their raw form as they did not show any group effect in the preliminary analysis (see Supplementary data).

Finally, we planned to verify by means of a correlational approach whether any in-group/out-group effects showed by the ANOVA would vary together with the individual implicit in-group preference as measured by the IAT D-Score.

The level of significance was set at *p* = 0.05. When appropriate, post-hoc tests were performed using Newman-Keuls method.

## Results

### Evaluation of the avatars

Firstly, we verified how much realistic and convincing the group of participants judged the avatar's movements. Results from a single-sample t-tests Bonferrori-corrected per multiple comparisons (final significance threshold, *p* = 0.05/2 = .025) showed REALISM ratings were higher than 50%, which would correspond to an intermediate judgment (50 mm) (mean REALISM = 68.9 ± 24.6 mm, t(1,13) = 2.87, *p_corr_* = .026). Conversely, DOUBT ratings (concerning whether in-/out-group avatar acted with different kinematics) were lower than the intermediate rating of 50% (50 mm) (mean DOUBT = 8.00 ± 21.5 mm, t(1,13) = −7.30, *p_corr_* < .001). With regard to individuals' judgments on their PERFORMANCE with the In-group/Out-group avatar, results from a dependent-sample t-test showed they did not differ (mean In-group PERFORMANCE = 50.79 ± 19.13 mm, mean Out-group PERFORMANCE = 56.50 ± 22.89 mm; t(1,13) = −1.07, *p* = .30), indicating that (at an explicit level) participants did not feel their performance depended on the partner's ethnic group.

The above mentioned results confirmed the assumption that participants judged the avatar's movements as realistic. Thus, we analyzed data from the human-avatar interaction as described in the methods. Namely, we included avatar's Group membership (In-group/Out-group) as within subject factor in the ANOVA.

### Interference effects during human-avatar interaction

In this section we report only the results concerning the ratio between Complementary and Imitative interactions in GAsynchr and MaxAp in order to focus on visuo-motor interference effects (see Supplementary data and Supplementary Tables S1 and S2 for the analyses on raw Accuracy, RTs, GAsynchr and MaxAp). For the sake of clarity, we separate the significant results linked to purely motor effects, which did not include the factor Group and were thus not linked to in-group/out-group modulations but rather depended on task constraints (*Non Group-related interference effects* paragraph), from in-group/out-group effects (*Effects of Group membership on visuo-motor interference* and *Correlation between Group membership interference effects and racial bias* paragraphs).

#### Non Group-related interference effects

All non Group-related significant results are reported in Table 1 and described below with reference to the significance of each post-hoc test.

***GAsynchr***. The ANOVA showed a significant main effect of Correction (Correction/No-correction) and a significant Correction (Correction/No-correction) x Grip-type (Power/Precision) interaction. Post-hoc analysis indicates interference was associated to higher synchrony in No-correction-Power grips as compared to the other conditions (all *p*s < .005). In particular, No-correction-Power grips was the only condition in which participants achieved higher synchrony in the Complementary condition with respect to the Imitative one (mean value lower than one, *p* = .03). This indicates that in the No-corrections-Power grip condition participants found it easier to synchronize with an avatar performing a precision grip (i.e. during complementary trials) vs. a power grip (i.e. during imitative trials). Importantly, this effect was independent from the partner's in-group/out-group membership.

***MaxAp***. The ANOVA on MaxAp showed a significant main effect of Interaction-type (Syn/JA) and an Interaction-type (Syn/JA) x Grip-type (Power/Precision) significant interaction. Post-hoc tests indicate that in Precision, but not in Power grips, interference was lower (i.e. MaxAp was smaller) when the task required to synchronize in time only (i.e. in Syn) with respect to when prediction of the avatar's goal was also required (i.e. in JA, all *p*s < .009). It is worth noting that the mean value in this latter condition (JA-Precision grip), however, was not significantly different than 1 (*p* = .08). Instead, this effect was strongly modulated by the Group factor (see in-group/out-group result section below).

#### Effects of Group membership on visuo-motor interference

***GAsynchr****.* No main effect nor interaction with the factor Group reached statistical significance.

**Figure 3:**
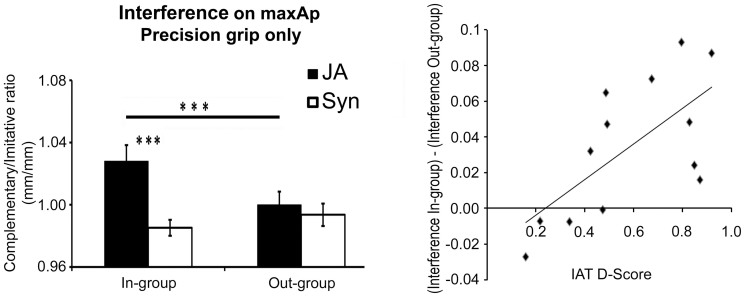
On the left: the analyses of visuo-motor interference on MaxAp data (interference index obtained dividing Complementary/Imitative trails). The graph shows the Partner x Interaction-type x Grip-type significant interaction (F(1,13) = 8.8, *p* = .011, partial η^2^ = .4) indicating that visuo-motor interference effects emerged only in Precision grips during JA interactions with the In-group. Error bars indicate s.e.m. (***) *p* < .001. On the right: the highly significant positive correlation (*r* = .67, *p* = .012) indicates that the difference between visuo-motor interference (Complementary/Imitative MaxAp, mm/mm) emerged with the In-group as compared to with the Out-group partner [(In-group visuo-motor interference) – (Out-group visuo-motor interference)] was higher in the participants with stronger bias.

***MaxAp****.* The ANOVA on MaxAp showed a significant Group (in-group/out-group) x Interaction-type (JA/Syn) interaction (F(1,13) = 5.21, *p* = .04; partial η^2^ = .29), and a significant Group x Interaction-type x Grip-type interaction (F(1,13) = 8.8*, p* = .011, partial η^2^ = .40). These interactions showed that visuo-motor interference emerged only in JA, only when interacting with the In-group avatar and only during Precision grips (all *p*s ≤ .001; JA–Precision grip–In-group vs. JA-Precision grip–Out-group, *d* = .78). As a matter of fact, In-group–JA–Precision grip was the only condition showing a Complementary/Imitative ratio higher than 1 (single-sample one-tailed t-test, *p* = .009, see Figure 3, left panel). This indicates that In-group–JA–Precision grip was the only condition in which visuo-motor interference emerged.

#### Correlation between Group membership interference effects and racial bias

In order to test whether the modulation of visuo-motor interference effects described above was linked to individual racial prejudices, we computed an index of visuo-motor interference with the In-group vs. Out-group partner. This index was obtained by subtracting for each participant the Out-Group from In-group interference effects (i.e. the Complementary/Imitative ratio in JA-Precision grip), and it was correlated with individual IAT D-scores.

The analysis showed a highly significant positive correlation (*r* = .67, *p* = .012; see Figure 3, right panel), indicating the higher the IAT D-score, the greater was the difference between visuo-motor interference with the In-group partner as compared to with the Out-group. This suggests that the categorization of the partner as an In-group/Out-group individual had an impact on visuo-motor interference. More specifically, visuo-motor interference on kinematics in JA conditions was higher for In-group than Out-group interactions only in participants with stronger group bias (see Figure 3, right panel). Note that analysis of Cook's distances revealed one participant as an outlier. Thus, the correlation analyses were performed on 13 out of the 14 participants.

## Discussion

Humans are extremely prone to classify others according to “Us vs. Them” categories^3^,^44^. Race represents a powerful cue to group membership, especially in the absence of other affiliation factors. In the present study, we expanded previous findings on the effect of racial membership in modulating the sensorimotor simulation triggered by observation of others' sensorimotor states by exploring i) whether face-to-face interactions with in-group vs. out-group individuals modulate specific kinematics features, and ii) whether these modulations are selectively linked to action prediction. One main result is that during face-to-face complementary motor interactions the social categorisation of virtual partners as in-group/out-group members modulates the recruitment of sensorimotor simulation of the partner's movements as indirectly indexed by visuo-motor interference. Moreover, this modulation is specific for conditions where making predictions about the partner's actions goals is required (i.e. in JA). Tellingly, this in-group/out-group modulation of simulation (as indexed by visuo-motor interference) strongly correlates with the individual degree of implicit in-group/out-group racial bias as measured by the Implicit Association Test.

The analysis of kinematics allowed us to monitor participants' behaviour during the interaction and verify whether they were on-line adapting to the avatar's movements, thus validating human-avatar interactive scenario as a reliable proxy to human-human joint actions in a controlled environment (see Figure 2, Supplementary data and Supplementary Fig. S1).

Results on maximum grip Aperture showed visuo-motor interference effects^25^ between self-executed actions and those observed in the partner emerged only during JA, and, crucially, only when interacting with an In-group avatar. That visuo-motor interference was evident during JA only highlights the close link between action simulation and action prediction and the subtle differences between these two processes^20^,^21^,^22^,^23^: indeed, sensorimotor simulation (indirectly indexed by visuo-motor interference) was recruited only when participants needed to predict the goals of the partner's movements in order to adapt to them, i.e. in JA. This evidence also extend previous studies showing that activity of the so-called fronto-parietal “mirror” network^7^ is recruited during the execution of complementary joint actions^45^ and that the observation of actions requiring a complementary response triggers “mirror” simulation of the observed actions before a complementary response is performed^46^. Finally, in-group/out-group modulation of visuo-motor interference was shown in precision grip kinematics only. This is in line with studies showing that an agent's goal^27^,^28^ and an agent's communicative intention specifically modulate precision but not power grip aperture^47^. This might be linked to evidence that functional connections between motor and premotor cortices during grasping are strong in precision and absent in power grip^34^ indicating fine-tuned on-line control of movement execution is required more in the former than in the latter. It is worth noting that during power grip contact points of the finger on the grasped object are also modulated by the agent's goal (see for instance^48^). As in our task contact points on the bottle were predetermined, they were not taken into consideration. Thus, significant effects were expected to emerge in precision grip only.

Being involved in complementary actions during JA influenced participants' movement execution only when interacting with an In-group avatar. Importantly, participants were able to achieve a comparable level of performance interacting with either the in-group or out-group partner. This is suggested by the analysis of GAsynchr which indicates that the in-group/out-group modulation of visuo-motor interference was not paralleled by an analogue in-group/out-group effect in interpersonal coordination performance. Thus, embodied sensorimotor simulation (as indirectly indexed here by visuo-motor interference) might be just one of the possible mechanisms leading to achieve interpersonal coordination. However, it might have a social connotation, as it might be a sign of perceived affiliation between interactive partners. That on-line adaptation to the partner's movement during corrections was equal with the In-group and with the Out-group partner (see Figure 2, Supplementary data and Supplementary Fig. S1) suggests racial bias does not lead to a general impairment in the ability to on-line coordinate with others but have a specific impact on sensorimotor simulation recruitment as indexed by visuo-motor interference. As suggested by Schmader and colleagues^49^, stereotypes might impact on performance (e.g. on athletic performance, see for instance^50^) through a modulation of working memory and of monitoring/prefrontal functioning. However, the purely sensory-motor nature of the visuo-motor interference effect described here rules out that such a general cause might have an impact on performance in the present case. Furthermore, the absence of in-group/out-group differences in accuracy and reaction times (see Supplementary data) also suggests that visuo-motor interference may not be accounted for by non-specific motivational/attentional factors. On the contrary, the significant effects emerged from MaxAp analyses suggest that during face-to-face joint actions racial stereotypes likely modulate the degree of sensorimotor simulation that may be crucial for automatically reading the partner's movements. Such simulation might be the basic mechanism that allows inter-individuals alignment in everyday life interactions (see also ref. 51). Accordingly, unconscious mimicry of others' postures and mannerisms during interactions^52^ may have the social scope of promoting affiliation^53^,^54^,^55^. Conversely, it has been suggested that the voluntary mimicry of out-group members may reduce racial bias^56^. Tellingly, the reduction of self-other distinction induced by body ownership illusions may also be an effective way to change and reduce negative implicit attitudes towards out-groups^5^,^6^; further research is needed to investigate whether the reinforcement of social bonds that arise during motor interactions^43^ might exert the same powerful modulation.

In conclusion, visuo-motor interference evoked by the observation of a partner's movements during face-to-face motor interactions is modulated by the partner's in-group/out-group membership. More specifically, our results suggest that embodied simulation might be shaped by cultural inter-individual differences^10^,^11^,^12^,^17^, since the in-group/out-group modulation of interactive kinematics varies according to individuals' racial bias. Although the absence of visuo-motor interference during interactions with the Out-group is reminiscent of the modulation exerted by racial bias on mirror-like responses in others' action and pain observation^8^,^9^,^10^,^11^,^12^, our results show that visuo-motor interference (and its in-group/out-group modulation) arises only when participants need to predict the partner's movements in order to adapt to them. Thus, our study suggests a close link between motor simulation and action prediction in interactive contexts^20^,^23^,^24^. Moreover, we expand previous studies on the impact of social variables on joint-action kinematics^32^,^43^,^57^: by highlighting that visuo-motor interference with the in-group partner was higher in biased participants only, our results indirectly suggest that the recruitment of predictive sensorimotor simulation during joint action might constitute the marker of perceived affiliation between interactive partners. Consequently, socially biased interactions might be rooted in a modulation of low-level sensorimotor mechanisms and indexed by movement kinematics.

## Supplementary Material

Supplementary Information Supplementary data and figures

## Figures and Tables

**Table 1:** All Non Group-related significant effects in GAsynchr and MaxAp on the Complementary/Imitative ratio (i.e. index of interference)

GAsynchr				
Effect	df	F	*p*	*Partial Eta-Squared*
Main effect of Correction	1,13	6.48	.024	.33
Correction x Grip-type	1,13	12.53	.004	.49

MaxAp				
Effect	df	F	*p*	*Partial Eta-Squared*
Main effect of Interaction- type	1,13	14.41	.002	.52
Interaction-type x Grip- type	1,13	6.76	.02	.34

## References

[b1] Kubota J. T., Banaji M. R. & Phelps E. A. The neuroscience of race. Nat. Neurosci. 15, 940–8 (2012). 22735516 10.1038/nn.3136 PMC3864590

[b2] Dunham Y., Baron A. S. & Banaji M. R. The development of implicit intergroup cognition. Trends Cogn. Sci. 12, 248–53 (2008). 18555736 10.1016/j.tics.2008.04.006

[b3] Amodio D. M. The social neuroscience of intergroup relations. Eur. Rev. Soc. Psychol. 19, 1–54 (2008).

[b4] Ito T. A. & Bartholow B. D. The neural correlates of race. Trends Cogn. Sci. 13, 524–531 (2009). 19896410 10.1016/j.tics.2009.10.002 PMC2796452

[b5] Maister L., Sebanz N., Knoblich G. & Tsakiris M. Experiencing ownership over a dark-skinned body reduces implicit racial bias. Cognition 128, 170–8 (2013). 23680793 10.1016/j.cognition.2013.04.002 PMC3750641

[b6] Peck T. C., Seinfeld S., Aglioti S. M. & Slater M. Putting yourself in the skin of a black avatar reduces implicit racial bias. Conscious. Cogn. 22, 779–787 (2013). 23727712 10.1016/j.concog.2013.04.016

[b7] Rizzolatti G. & Craighero L. The mirror-neuron system. Annu. Rev. Neurosci. 27, 169–92 (2004). 15217330 10.1146/annurev.neuro.27.070203.144230

[b8] Molnar-Szakacs I., Wu A. D., Robles F. J. & Iacoboni M. Do you see what I mean? Corticospinal excitability during observation of culture-specific gestures. PloS One 2, e626 (2007). 17637842 10.1371/journal.pone.0000626 PMC1913205

[b9] Désy M. C. & Théoret H. Modulation of motor cortex excitability by physical similarity with an observed hand action. PloS One 2, e971 (2007). 17912350 10.1371/journal.pone.0000971 PMC1989142

[b10] Gutsell J. N. & Inzlicht M. Empathy constrained: Prejudice predicts reduced mental simulation of actions during observation of outgroups. J. Exp. Soc. Psychol. 46, 41–845 (2010).

[b11] Avenanti A., Sirigu A. & Aglioti S. M. Racial bias reduces empathic sensorimotor resonance with other-race pain. Curr. Biol. 20, 1018–1022 (2010). 20537539 10.1016/j.cub.2010.03.071

[b12] Azevedo R. T. *et al.* Their pain is not our pain: Brain and autonomic correlates of empathic resonance with the pain of same and different race individuals. Hum. Brain. Mapp. 34, 3168–81 (2013). 22807311 10.1002/hbm.22133 PMC6870096

[b13] Liew S. L., Han S. & Aziz-Zadeh L. Familiarity modulates mirror neuron and mentalizing regions during intention understanding. Hum. Brain Mapp. 32, 1986–97 (2011). 20882581 10.1002/hbm.21164 PMC6870503

[b14] Earls H. A., Englander Z. A. & Morris J. P. Perception of race-related features modulates neural activity associated with action observation and imitation. Neuroreport 24, 410–3 (2013). 23571693 10.1097/WNR.0b013e328360a168

[b15] Losin E. A., Iacoboni M., Martin A., Cross K. A. & Dapretto M. Race modulates neural activity during imitation. Neuroimage 59, 3594–603 (2012). 22062193 10.1016/j.neuroimage.2011.10.074 PMC3909702

[b16] Pavan G., Dalmaso M., Galfano G. & Castelli L. Racial group membership is associated to gaze-mediated orienting in Italy. PLoS One 6, e25608 (2011). 21991323 10.1371/journal.pone.0025608 PMC3186779

[b17] Chiao J. Y. & Mathur V. A. Intergroup empathy: How does race affect empathic neural responses? Curr. Biol. 20, 478–480 (2010). 10.1016/j.cub.2010.04.001 20541493

[b18] Sebanz N. & Knoblich G. Prediction in Joint Action: What, When, and Where. Top. Cogn. Sci. 1, 353–367 (2009). 25164938 10.1111/j.1756-8765.2009.01024.x

[b19] Knoblich G. & Jordan J. S. Action coordination in groups and individuals: learning anticipatory control. J. Exp. Psychol. Learn. Mem. Cogn. 29, 1006–16 (2003). 14516231 10.1037/0278-7393.29.5.1006

[b20] Kilner J. M., Friston K. J. & Frith C. D. The mirror-neuron system: a Bayesian perspective. Neuroreport 18, 619–23 (2007). 17413668 10.1097/WNR.0b013e3281139ed0

[b21] Urgesi C. *et al.* Simulating the future of actions in the human corticospinal system. Cereb. Cortex, 20, 2511–21 (2010). 20051359 10.1093/cercor/bhp292

[b22] Avenanti A., Annella L., Candidi M., Urgesi C. & Aglioti S. M. Compensatory plasticity in the action observation network: virtual lesions of STS enhance anticipatory simulation of seen actions. Cereb Cortex 23, 570–80 (2013). 22426335 10.1093/cercor/bhs040

[b23] Aglioti S. M., Cesari P., Romani M. & Urgesi C. Action anticipation and motor resonance in elite basketball players. Nat. Neurosci. 11, 1109–1116 (2008). 19160510 10.1038/nn.2182

[b24] Candidi M., Sacheli L. M., Mega I. & Aglioti S. M. Somatotopic mapping of piano fingering errors in sensorimotor experts: tms studies in pianists and visually trained musically naïves. Cereb. Cortex. 24, 435–43 (2014). 23064109 10.1093/cercor/bhs325

[b25] Kilner J. M., Paulignan Y. & Blakemore S. J. An interference effect of observed biological movement on action. Curr. Biol. 13, 522–525 (2003). 12646137 10.1016/s0960-9822(03)00165-9

[b26] Blakemore S. J. & Frith C. The role of motor contagion in the prediction of action. Neuropsychologia 43, 260–7 (2005). 15707910 10.1016/j.neuropsychologia.2004.11.012

[b27] Ansuini C., Santello M., Massaccesi S. & Castiello U. Effects of end-goal on hand shaping. J. Neurophysiol. 95, 2456–2465 (2006). 16381806 10.1152/jn.01107.2005

[b28] Ansuini C., Giosa L., Turella L., Altoè G. & Castiello U. An object for an action, the same object for other actions: effects on hand shaping. Exp. Brain Res. 185, 111–119 (2008). 17909766 10.1007/s00221-007-1136-4

[b29] Georgiou I., Becchio C., Glover S. & Castiello U. Different action patterns for cooperative and competitive behaviour. Cognition 102, 415–33 (2007). 16516188 10.1016/j.cognition.2006.01.008

[b30] Becchio C., Sartori L., Bulgheroni M. & Castiello U. The case of Dr. Jekyll and Mr. Hyde: a kinematic study on social intention. Conscious. Cogn. 17, 557–64 (2008a). 17446089 10.1016/j.concog.2007.03.003

[b31] Becchio C., Sartori L., Bulgheroni M. & Castiello U. Both your intention and mine are reflected in the kinematics of my reach-to-grasp movement. Cognition 106, 894–912 (2008b 17585893 10.1016/j.cognition.2007.05.004

[b32] Becchio C., Sartori L. & Castiello U. Toward You: The Social Side of Actions. Curr. Dir. Psychol. Science 19, 183–188 (2010).

[b33] Muir R. B. & Lemon R. N. Corticospinal neurons with a special role in precision grip. Brain Res. 261, 312–6 (1983). 6831213 10.1016/0006-8993(83)90635-2

[b34] Davare M., Lemon R. & Olivier E. Selective modulation of interactions between ventral premotor cortex and primary motor cortex during precision grasping in humans. J. Physiol. 586, 2735–2742 (2008). 18403420 10.1113/jphysiol.2008.152603 PMC2536583

[b35] Castiello U. The neuroscience of grasping. Nat. Rev. Neurosci. 6, 726–36 (2005). 16100518 10.1038/nrn1744

[b36] Grafton S. T. The cognitive neuroscience of prehension: recent developments. Exp. Brain Res., 204, 475–491 (2010). 20532487 10.1007/s00221-010-2315-2 PMC2903689

[b37] Brass M., Bekkering H. & Prinz W. Movement observation affects movement execution in a simple response task. Acta Psychol (Amst) 106, 3–22 (2001). 11256338 10.1016/s0001-6918(00)00024-x

[b38] Bortoletto M., Baker K. S., Mattingley J. B. & Cunnington R. Visual-motor interactions during action observation are shaped by cognitive context. J. Cogn. Neurosci. 25, 1794–806 (2013a). 23767924 10.1162/jocn_a_00431

[b39] Bortoletto M., Mattingley J. B. & Cunnington R. Effects of context on visuomotor interference depends on the perspective of observed actions. PLoS One 8, e53248 (2013b). 23301050 10.1371/journal.pone.0053248 PMC3536761

[b40] Briggs G. G. & Nebes R. D. Patterns of hand preference in a student population. Cortex 11, 230–8 (1975). 1204363 10.1016/s0010-9452(75)80005-0

[b41] Greenwald A. G., McGhee D. E. & Schwartz J. L. Measuring individual differences in implicit cognition: the implicit association test. J. Pers. Soc. Psychol. 74, 1464–1480 (1998). 9654756 10.1037//0022-3514.74.6.1464

[b42] Greenwald G. A., Nosek B. A. & Banaji M. R. Understanding and Using the Implicit Association Test: I. An Improved Scoring Algorithm. J. Pers. Soc. Psychol. 85, 197–216 (2003). 12916565 10.1037/0022-3514.85.2.197

[b43] Sacheli L. M., Candidi M., Pavone E. F., Tidoni E. & Aglioti S. M. And yet they act together: interpersonal perception modulates visuo-motor interference and mutual adjustments during a joint-grasping task. PLoS One 7, e50223 (2012). 23209680 10.1371/journal.pone.0050223 PMC3509140

[b44] Tajfel H. Human Groups and Social Categories. Cambridge [Cambridgeshire]: Cambridge University Press., 132–134 (1981).

[b45] Newman-Norlund R. D., van Schie H. T., van Zuijlen A. M. & Bekkering H. The mirror neuron system is more active during complementary compared with imitative action. Nat. Neurosci. 10, 817–8 (2007). 17529986 10.1038/nn1911

[b46] Sartori L., Bucchioni G. & Castiello U. When emulation becomes reciprocity. Soc. Cogn. Affect. Neurosci. 8, 662–9 (2013). 22490925 10.1093/scan/nss044 PMC3739911

[b47] Sartori L., Becchio C., Bara B. G. & Castiello U. Does the intention to communicate affect action kinematics? Conscious. Cogn. 18, 766–72 (2009). 19632134 10.1016/j.concog.2009.06.004

[b48] Sartori L., Straulino E. & Castiello U. How objects are grasped: the interplay between affordances and end-goals. PLoS One 6, e25203 (2011). 21980396 10.1371/journal.pone.0025203 PMC3182194

[b49] Schmader T., Johns M. & Forbes C. An Integrated Process Model of Stereotype Threat Effects on Performance. Psychol. Rev. 115, 336–356 (2008). 18426293 10.1037/0033-295X.115.2.336 PMC2570773

[b50] Stone J., Lynch C. I., Sjomeling M. & Darley J. M. Stereotype threat effects on Black and White athletic performance. Journal of personality and social psychology, 77, 1213 (1999).

[b51] Colling L. J., Knoblich G. & Sebanz N. How does “mirroring” support joint action? Cortex 49, 2964–5 (2013). 23962894 10.1016/j.cortex.2013.06.006

[b52] Chartrand T. L. & Bargh J. A. The chameleon effect: The perception-behavior link and social interaction. J. Pers. Soc. Psychol. 76, 893–910 (1999). 10402679 10.1037//0022-3514.76.6.893

[b53] van Baaren R. B., Holland R. W., Kawakami K. & van Knippenberg A. Mimicry and prosocial behavior. Psychol. Sci. 15, 71–74 (2004). 14717835 10.1111/j.0963-7214.2004.01501012.x

[b54] van Baaren R., Janssen L., Chartrand T. & Dijksterhuis A. Where is the love? The social consequences and moderators of mimicry in humans. Proc. R. Soc. B. 364, 2381–2389 (2009). 10.1098/rstb.2009.0057 PMC2865082 19620109

[b55] Lakin J. & Chartrand T. L. Using nonconscious behavioral mimicry to create affiliation and rapport. Psychol. Sci. 14, 334–339 (2003). 12807406 10.1111/1467-9280.14481

[b56] Inzlicht M., Gutsell J. N. & Legault L. Mimicry reduces racial prejudice. J. Exp. Soc. Psyc. 48, 361–365 (2012).

[b57] Sacheli L. M., Tidoni E., Pavone E. F., Aglioti S. M. & Candidi M. Kinematics fingerprints of leader and follower role-taking during cooperative joint actions. Exp. Brain Res. 226, 473–86 (2013). 23503771 10.1007/s00221-013-3459-7

