# Supplementary material for: Prejudiced interactions: implicit racial bias reduces predictive simulation during joint action with an out-group avatar
Source: Sci Rep. 2015 Feb 17;5:8507. doi: 10.1038/srep08507 (PMC5389129; doi:10.1038/srep08507)
Supplement: Supplementary Information — Supplementary data and figures [file srep08507-s1.pdf]

Prejudiced interactions: implicit racial bias reduces predictive simulation during joint action with an out-group avatar

Lucia Maria Sacheli[1,2], Andrea Christensen[3,4], Martin A Giese[3,4], Nick Taubert[3,4], Enea F Pavone[1,2], Salvatore M Aglioti [1,2], Matteo Candidi[1,2]

1. Department of Psychology, Sapienza University of Rome, Italy.
2. IRCCS, Fondazione Santa Lucia, Rome, Italy.
3. Section Computational Sensomotorics, Department of Cognitive Neurology, Hertie Institute for Clinical Brain Research.
4. Centre for Integrative Neuroscience, University Clinic Tübingen, Germany.

Supplementary data

Reaction Times, Accuracy, GAsynchrony and MaxAp individual means were preliminarily entered in separate within-subject ANOVAs having Group (In-/Out-group) x Action-type (Complementary/Imitative) x Correction (Correction/No-correction) x Interaction-type (Syn/JA) x Grip-type (Power/Precision grip) as within subjects factors.

For the sake of clarity, we separate here significant results linked to in-group/out-group effects (paragraph *Group membership effects*) from purely motor significant effects which did not include the factor Group and were thus not linked to in-group/out-group effects but rather depended on task constrains (paragraph *Non Group-related effects*). With regard to grasping Asynchrony (GAsynchr) and maximum grip aperture (MaxAp), all significant results are reported in Table S1 and Table S2 and described below with reference to the significance of each post-hoc test.

Non Group-related effects.

Accuracy. Overall, participants were highly accurate in performing the task (mean Accuracy = 95% ± 2.3). Since one experimental condition (i.e. Out-group–Complementary–NoCorrection-JA-PowerGrip) was at ceiling, data did not allow for a factorial ANOVA. However, In-group vs. Out-group participants' mean accuracy was not statistically different (dependent-samples t-test: mIN-GROUP = 95.2 ± 2.4 %, mOUT-GROUP = 95.87 ± 2.3 %, t(1,13) = -1.90, p = .078).

Reaction Times. The ANOVA on RTs showed a significant main effect of Interaction-type (F(1,13) = 55.09, p < .001, partial η^2 = .8), indicating JA required longer RTs than Syn. The Interaction-type x Correction interaction was also significant (F(1,13) = 5.04, p = .043, partial η^2 = .28), and indicated RTs in Syn were significantly faster during observation of a Correction than a No-correction clip (p = .03). Importantly, RTs did not show any main effect or significant interaction with Group, indicating movement preparation prior to the proper joint-execution was not influenced by the avatar's group membership. This also suggests an equal distribution of participants' attention when interacting with either the In-group or the Out-group avatar. In this regard, it is worth noting that during the on-line interactive task described here participants could not plan their own movements when receiving the auditory instructions, because they were required to on-line adapt their movements to the partner's ones in time (Syn) or both in time and space (JA). Thus, changes in RTs seem unlikely to reflect predictive processes but rather attentional ones.

GAsynchr. See Supplementary Table S1 for a description of all significant effects. The ANOVA on GAsynchr showed a significant main effect of Correction, indicating

it was easier for participants to synchronize with the avatars in No-correction clips. Moreover, the Action-type x Correction and Action-type x Correction x Grip-type significant interactions showed that, regardless the Group, synchronization in Correction clips was more difficult in Complementary as compared to Imitative actions (Correction-Complementary vs. Correction–Imitative, p = .001) and that this effect was significant only when participants had to change from a Precision to a Power grip (Complementary–Correction–Power grip vs. Imitative–Correction–Power grip, p = .016). Moreover, the Action-type x Correction x Grip-type significant interaction showed that, during No-Correction, GAsynchr was better during Complementary as compared to Imitative Power grips (p = .022).

**Grasping Asynchrony**				
**Effect**	**df**	**F**	***p***	***Partial Eta-Squared***
Main effect of Correction	1,13	60.09	< .001	.82
Interaction-type x Correction	1,13	6.12	.028	.32
Action-type x Correction	1,13	10.55	.006	.44
Action-type x Correction x Grip-type	1,13	15.07	.002	.54
**Group x Interaction-type x Correction**	**1,13**	**4.93**	**.045**	**.27**
**Group x Interaction-type x Correction x Grip-type**	**1,13**	**4.74**	**.048**	**.27**

Supplementary Table S1. All significant effects emerged from the ANOVA on Grasping Asynchrony (GAsynchr). **In bold**, significant interaction with the within-factor Group, i.e. In-group/Out-group effects.

MaxAp. See Supplementary Table S2 for a description of all significant effects. The ANOVA on MaxAp showed a significant main effect of Grip-type indicating that, as expected, MaxAp was larger in Power compared to Precision grips. Moreover, it showed the significant main effects of Correction and Interaction-type, indicating that MaxAp was larger in Correction compared to No-corrections and during JA compared to Syn, probably reflecting participants' attempt to increase the safety margin in Corrections as compared to No-corrections and in JA as compared to Syn. Post-hoc tests on the two-way Interaction-type x Correction, Correction x Grip-type and Interaction-type x Grip-type significant interactions further specified that: i) MaxAp in Corrections was larger than in No-corrections only during JA ($p < .001$), ii) MaxAp in Corrections was larger than in No-corrections only for Precision grips ($p < .001$), and iii) MaxAp during JA was larger than during Syn only for Precision grips ($p < .001$). Finally, the three-way Interaction-type x Correction x Grip-type significant interaction additionally clarified these effects by showing that, in Precision grips only, MaxAp was larger in Corrections as compared to No-corrections during both JA ($p < .001$) and Syn ($p = .024$), but MaxAp in Correction-JA was highly significantly larger than in Correction-Syn ($p < .001$). As shown by the Supplementary Figures S1 and Figure 2 in the main text, the above mentioned effects demonstrate that participants actually changed their movement in response to the avatars' correction in JA. Indeed, in this condition their MaxAp in Precision grips gets closer to the MaxAp typical of Power grips. This modulation was only found in Precision grips probably due to the more accurate and complex nature of precision grip planning and control (see main text). Importantly, this interaction was not modulated by in-group/out-group membership of the avatar. In other words, these results show that on-line adjustments were present when participants were interacting both with the in-group and with the

out-group partner. These results support the claim that participants were equally engaged in the interaction with both the in-group and the out-group virtual partner. Indeed they on-line adapted to both of them, suggesting that group-related modulation of visuo-motor interference (see main text) may not be accounted for by general motivational/attentional factors.

**Maximum grip aperture**				
**Effect**	**df**	**F**	***p***	***Partial Eta-Squared***
Main effect of Correction	1,13	75.0	<.001	.85
Main effect of Interaction-type	1,13	61.8	<.001	.83
Main effect of Grip-type	1,13	149.1	<.001	.91
Interaction-type x Clip-type	1,13	37.8	<.001	.74
Interaction-type x Grip-type	1,13	105.8	<.001	.89
Correction x Grip-type	1,13	60.5	<.001	.82
Interaction-type x Correction x Grip-type	1,13	31.6	<.001	.70
Action-type x Interaction-type	1,13	13.3	.003	.50
**Main effect of Group**	**1,13**	**5.1**	**.042**	**.28**
**Group x Action-type x Interaction-type x Grip-type**	**1,13**	**8.5**	**.021**	**.39**

Supplementary Table S2. All significant effects on maximum grip aperture. **In bold**, significant effects including the within factor Group, i.e. In-group/Out-group effects.

Lastly, Action-type x Interaction-type significant interaction showed that, overall, MaxAp in Complementary actions was smaller than in Imitative actions only during Syn (*p* = .006), but not during JA. This effect was strongly modulated by the Group

(see below and paragraph *Effects of Group membership on visuo-motor interference* in the main text).

Group membership effects.

Accuracy and Reaction Times. No in-group-out-group significant effect emerged.

GAsynchr. See Table S1 for a description of all significant effects. The Group x Interaction-type x Correction significant interaction showed GAsynchr in Corrections tended to be better during JA as compared to Syn only when interacting with the In-group partner (p = .008, d = .61, see Supplementary Figure S2). The Group x Interaction-type x Correction x Grip-type significant interaction suggested this facilitation in JA Corrections with the Ingroup was stronger during Power grips (In-group– JA –Correction–Power grip vs. In-group-Syn-Correction-Power grip p = .048, d = .64). Crucially, this different performance in Corrections during JA as compared to Syn was not present during interactions with the Out-group (p = .579).

MaxAp. See Table S2 for a description of all significant effects. The ANOVA on MaxAp showed a significant main effect of Group, suggesting participants' MaxAp was generally larger when they interacted with the Out-group partner. This effect was explained by the four-way Group x Action-type x Interaction-type x Grip-type significant interaction, showing that only during precision grips: i) participants' MaxAp during Imitative–JA was significantly smaller when interacting with the In-

group partner than with the Out-group (Precision grip only *p* = .001, *d* = .35) (note that the aperture shown by the in- and out-group avatar is identical), and crucially, ii) that MaxAp during JA interactions with the In-group partner only was significantly larger in Complementary as compared to Imitative actions (Precision grips only, *p* < .001, *d* = .45) (this shows that when one is strongly relying on predictions of the in-group avatar's goal he tends to simulate the avatar's movement and show strong interference effects), and significantly smaller in Complementary compared to Imitative actions during Syn (Precision grips only, *p* = .043, *d* = .19) (this shows that when synchronization may be achieved without relying on goal predictions, simulation of complementary movement is not at play). Overall these effects show that participants' kinematics were more strongly influenced by the avatar's ones when the avatar belonged to their own group.

Supplementary Figures.

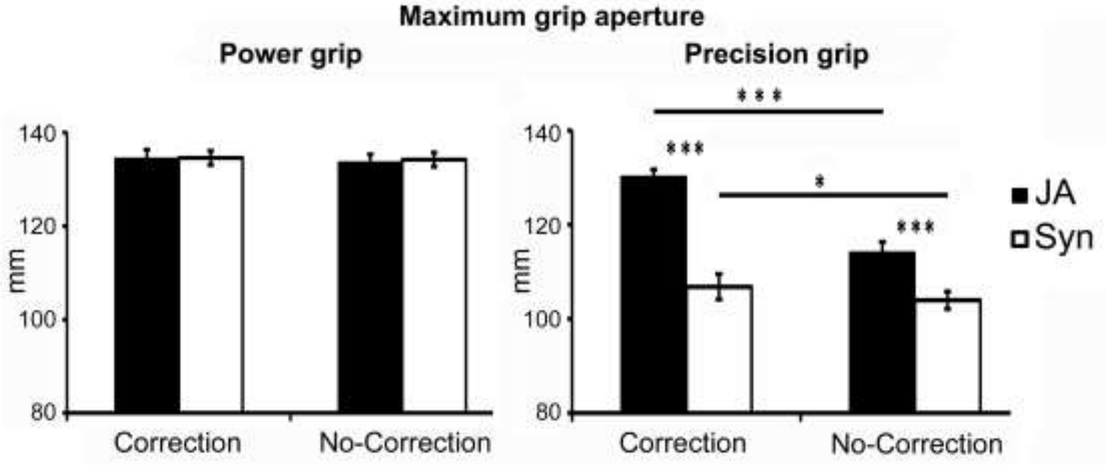

Supplementary Figure S1. The figure illustrates the significant Interaction-type x Correction x Grip-type interaction ($F(1,13) = 31.6$, $p < .001$, partial $\eta^2 = .7$) emerged from the ANOVA on MaxAp data. For the sake of simplicity, we did not report the significance of the comparison between Power and Precision grips in all conditions (all *p*s < .001 except for JA-

Corrections where Precision vs. Power grips, p = .012). Error bars indicate s.e.m. (***) $p <$ .001, (**) $p < .01$, (*) $p < .05$.

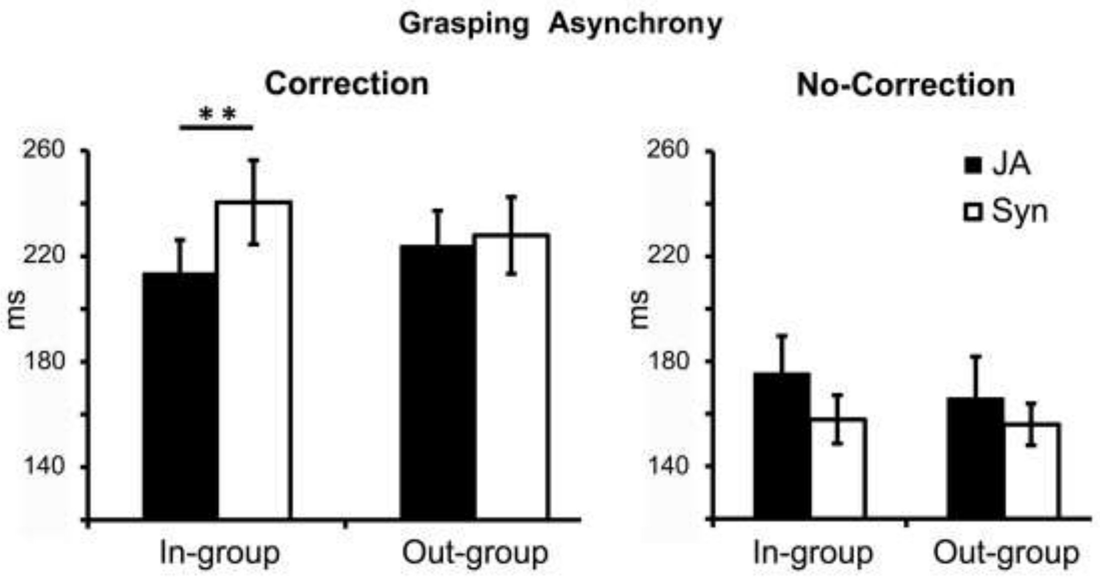

Supplementary Figure S2. The figure illustrates the Group x Interaction-type x Correction significant interaction (F(1,13) = 4.93, p = .045, partial η^2 = .27) showed by data on GAsynchr. For the sake of simplicity, we did not explicitly report in the figure the significance of the comparison between Correction and No-correction (all *p*s < .001, as also suggested by the significant main effect). Error bars indicate s.e.m.

(**) = $p < .01$.
